# The Use of Chimeric Virus-like Particles Harbouring a Segment of Hantavirus Gc Glycoprotein to Generate a Broadly-Reactive Hantavirus-Specific Monoclonal Antibody

**DOI:** 10.3390/v6020640

**Published:** 2014-02-07

**Authors:** Aurelija Zvirbliene, Indre Kucinskaite-Kodze, Ausra Razanskiene, Rasa Petraityte-Burneikiene, Boris Klempa, Rainer G. Ulrich, Alma Gedvilaite

**Affiliations:** 1 Vilnius University Institute of Biotechnology, V.A. Graiciuno 8, Vilnius LT-02241, Lithuania; E-Mails: indre.kodze@bti.vu.lt (I.K.-K.); ausra.razanskiene@bti.vu.lt (A.R.); rasa.burneikiene@bti.vu.lt (R.P.-B.); alma.gedvilaite@bti.vu.lt (A.G.); 2 Institute of Medical Virology, Helmut-Ruska-Haus, Charité Medical School, Berlin 10117, Germany; E-Mail: boris.klempa@charite.de; 3 Institute of Virology, Slovak Academy of Science, Bratislava 84505, Slovakia; 4 Institute for Novel and Emerging Infectious Diseases, Friedrich-Loeffler-Institut, Federal Research Institute for Animal Health, Südufer 10, Greifswald-Insel Riems 17493, Germany; E-Mail: Rainer.Ulrich@fli.bund.de

**Keywords:** hantavirus, Gc glycoprotein, monoclonal antibody, yeast expression system, chimeric virus-like particles

## Abstract

Monoclonal antibodies (MAbs) against viral glycoproteins have important diagnostic and therapeutic applications. In most cases, the MAbs specific to viral glycoproteins are raised against intact virus particles. The biosynthesis of viral glycoproteins in heterologous expression systems such as bacteria, yeast, insect or mammalian cells is often problematic due to their low expression level, improper folding and limited stability. To generate MAbs against hantavirus glycoprotein Gc, we have used initially a recombinant yeast-expressed full-length Puumala virus (PUUV) Gc protein. However, this approach was unsuccessful. As an alternative recombinant antigen, chimeric virus-like particles (VLPs) harboring a segment of PUUV Gc glycoprotein were generated in yeast *Saccharomyces cerevisiae*. A 99 amino acid (aa)-long segment of Gc protein was inserted into the major capsid protein VP1 of hamster polyomavirus at previously defined positions: either site #1 (aa 80–89) or site #4 (aa 280–289). The chimeric proteins were found to self-assemble to VLPs as evidenced by electron microscopy. Chimeric VLPs induced an efficient insert-specific antibody response in immunized mice. Monoclonal antibody (clone #10B8) of IgG isotype specific to hantavirus Gc glycoprotein was generated. It recognized recombinant full-length PUUV Gc glycoprotein both in ELISA and Western blot assay and reacted specifically with hantavirus-infected cells in immunofluorescence assay. Epitope mapping studies revealed the *N*-terminally located epitope highly conserved among different hantavirus strains. In conclusion, our approach to use chimeric VLPs was proven useful for the generation of virus-reactive MAb against hantavirus Gc glycoprotein. The generated broadly-reactive MAb #10B8 might be useful for various diagnostic applications.

## 1. Introduction

*Hantavirus* is a genus of the family *Bunyaviridae*, containing more than 20 different hantavirus species [1]. Hantaviruses with pathogenicity for human are carried by rodent reservoirs and are mainly transmitted to humans by inhalation of aerosols derived from excreta of infected rodents [2]. In addition, during the previous years a large number of novel shrew-, mole- and bat-borne, hantaviruses of unknown pathogenicity for human has been discovered [3]. The hantavirus virion contains three genome segments S, M and L, that encode the nucleocapsid (N) protein, glycoprotein precursor (GPC) and RNA-dependent RNA-polymerase, respectively [4]. GPC is a polyprotein of 1133–1158 amino acid (aa) residues in length [5]. Cotranslational cleavage of GPC at a site *C*-terminal to a conserved WAASA sequence by the cellular signal peptidase complex gives rise to glycoproteins Gn and Gc (previously known as G1 and G2, respectively) [6]. The hantavirus glycoproteins are responsible for virus attachment and entry into the host cells and are considered to be a major determinant of virus pathogenicity [5].

The viral RNA segments are associated with the nucleocapsid protein to form ribonucleoprotein (RNP) complexes [7]. The hantavirus Gn and Gc cytoplasmic tails mediate the interaction to the RNP and virion assembly [8,9,10]. The Gn and Gc proteins form a spike complex, which is located on the outer surface of the virion [11,12]. Each spike contains four molecules of both glycoproteins [13]. Virus-membrane fusion activity has been associated with Gc through the identification of a fusion peptide. The aa sequence of this peptide has remarkable conservation within representatives of the family *Bunyaviridae* [14]. 

Hantavirus infection in humans can cause two diseases with some similarities in their symptoms, hemorrhagic fever with renal syndrome (HFRS) and hantavirus cardiopulmonary syndrome (HCPS) [15,16,17]. The infection induces a strong humoral immune response that can be assessed by detecting virus-specific IgM or IgG antibodies. After the onset of the acute phase of hantavirus infection both IgM and IgG antibodies can be detected that react with hantavirus N protein, which represents the major target antigen of hantavirus-specific humoral immune response [18,19,20]. In contrast, antibodies against Gn and Gc appear later during the progress of disease [21]. For the serologic diagnosis of hantavirus infection, different assay formats such as indirect and capture ELISA, immunoblot test, immunochromatografic assay and indirect imunofluorescence assays using hantavirus-infected cells have been proven useful [22,23,24]. The majority of serologic tests are based on the use of recombinant proteins, mainly hantavirus N proteins expressed either in *E. coli*, yeast, or insect cells [25,26,27,28]. There are several reports on the heterologous expression of hantavirus Gn and Gc proteins in *E. coli*, yeast, insect and mammalian cell expression systems and their use for serologic detection of hantavirus infections and as potential vaccines [19,29,30,31,32]. In addition, Gn and Gc glycoproteins of different hantavirus strains have been expressed using recombinant vaccinia viruses [33,34,35,36].

Monoclonal antibodies (MAbs) raised against hantavirus antigens are widely used as diagnostic tools for hantavirus infection, either for the detection of virus-specific antibodies by different capture ELISA formats [23,26,37,38] or a direct detection of viral antigens in infected cell cultures or tissues [39,40]. In previous studies, several collections of recombinant and monoclonal antibodies specific to hantavirus glycoproteins have been described. The majority of these MAbs have been raised against an intact virus by immunisation and further selected for their reactivity with viral glycoproteins [41,42,43,44]. Alternatively, recombinant antibodies have been generated using B cells from naturally hantavirus-infected patients [45,46]. In one report glycoprotein-specifc MAbs were raised against *E. coli*-derived fragments (aa 647–886 and aa 829–1138) of Andes virus (ANDV) Gc protein and used for detection of ANDV antigens in urine samples [30].

Active immunisation with recombinant glycoproteins or glycoprotein-encoding DNA vaccine constructs have demonstrated their protective potential in animal models [47,48]. In addition, passive transfer of polyclonal sera to Hantaan virus (HTNV) or ANDV can passively protect hamsters and rats from challenge with hantaviruses [48,49,50]. Neutralizing hantavirus glycoprotein-specific MAbs derived either from mouse or human, have been demonstrated to passively protect from subsequent challenge with HTNV, Seoul virus (SEOV), and Puumala virus (PUUV) in rodent models [48,51,52,53,54,55,56,57,58]. The passive immunization of primates with neutralizing MAbs to PUUV protects them from subsequent PUUV challenge [59]. At present, there have been no published reports of controlled clinical trials of immunotherapy for HFRS or HCPS. However, a HTNV Gc-specific neutralizing MAb administered up to 4 days after virus challenge cured hamsters of infection [45]. These initial data suggest that a post-exposure prophylaxis treatment mode with hantavirus-neutralizing antibodies may be effective, as shown for other viral diseases such as rabies, hepatitis A and B, and varicella zoster viruses [60].

As hantavirus Gc glycoprotein represents a target for neutralizing antibodies and an important antigen for serologic tests [20,46], there is a need in efficient technologies for production of recombinant Gc glycoprotein derivatives. However, previous studies suggest that the biosynthesis of viral glycoproteins in heterologous expression systems is often problematic due to low expression level, improper folding and limited protein stability [61,62].

In the current study, we attempted to generate Gc-specific MAbs by using an entire hantavirus Gc protein and alternatively by using recombinant virus-like particles (VLPs) harbouring a selected hantavirus Gc protein segment. For the insertion into VLPs, we selected a 99 aa-long segment of PUUV-Gc protein spanning aa 880–978 of PUUV GPC that contained target epitopes for neutralizing antibodies [20,46]. We have demonstrated that the inserted PUUV-Gc segment was exposed on the surface of VLPs and elicited formation of Gc-specific antibodies.

## 2. Results

### 2.1. Production of Recombinant Full-Length PUUV-Gc Protein in Yeast S. cerevisiae and Generation of Monoclonal Antibodies

**Figure 1:**
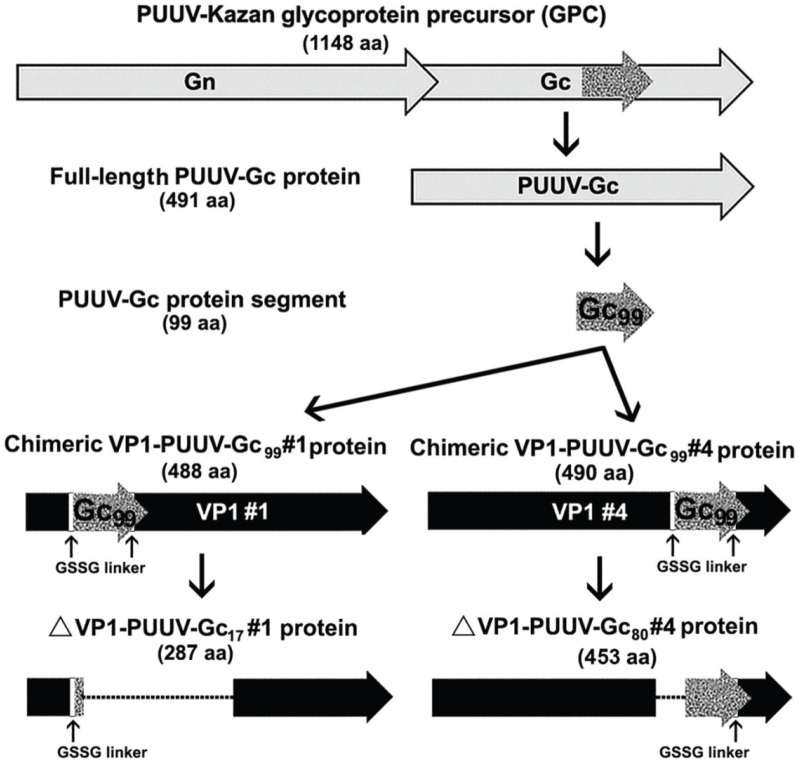
Schematic representation of Puumala virus (PUUV)-Gc constructs expressed in yeast. To produce chimeric virus-like particles (VLPs), PUUV-Gc_99_ segment (aa 880–978 of glycoprotein precursor (GPC)) was inserted into hamster polyomavirus VP1 protein between aa 80–89 in VP1-PUUV-Gc_99_#1 construct and between aa 280–289 in VP1-PUUV-Gc_99_#4 construct. For epitope mapping, two additional constructs were generated harbouring either aa 880–896 of GPC between aa 80–220 of VP1 protein (construct ΔVP1-PUUV-Gc17 #1) or aa 899–978 of GPC between aa 269–289 of VP1 protein (construct ΔVP1-PUUV-Gc80 #4).

*N*-terminally hexahistidin-tagged full-length PUUV Kazan Gc (PUUV-Gc) protein (Figure 1) was expressed in *S. cerevisiae* strain 8188 4D. The analysis of yeast cell lysate by sodium dodecylsulfate polyacrylamide gel electrophoresis (SDS-PAGE) demonstrated that recombinant PUUV-Gc protein is found predominantly in the insoluble fraction (data not shown). The identity of PUUV-Gc protein was confirmed by its immunoreactivity with anti-tetra histidine MAb and rabbit polyclonal anti-PUUV antibodies (data not shown). 

**Figure 2:**
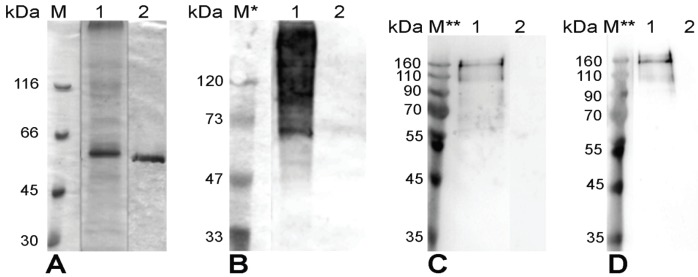
SDS-PAGE (**A**) and Western blot analysis (**B**–**D**) of purified full-length PUUV-Gc protein (lane 1) with an ubiquitin-specific polyclonal antibody (**B**) and the MAbs of clones #1F5 (**C**) and #7A12 (**D**) raised against yeast-expressed, His-tagged entire PUUV-Kazan Gc protein. Purified PUUV Vranica/Hällnäs N protein (lane 2) was used as a negative control. Lanes M, M * and M **, Prestained Protein Molecular Weight Markers (UAB, “Thermo Fisher Scientific Baltics”, Vilnius, Lithuania).

The His-tagged PUUV-Gc protein was purified from the insoluble fraction of yeast cell lysate using nickel chelate affinity chromatography (Figure 2A, lane 1). All disruption and purification steps were performed in the presence of strong denaturing agents (guanidinium hydrochloride followed by urea) and by adding protease inhibitors to minimize the risk of proteolytic degradation. Purified PUUV-Gc protein was used to immunize mice in order to generate Gc-specific MAbs. Two stable hybridoma clones (#1F5 and #7A12) producing MAbs of IgG isotype reactive with recombinant PUUV-Gc protein in ELISA were obtained. However, Western blot analysis of recombinant purified PUUV-Gc protein with the MAbs #1F5 and #7A12 revealed an untypical immunostaining pattern with specifically detected protein bands larger than 55 kDa and absence of protein bands corresponding to the theoretical molecular size of PUUV-Gc protein (Figure 2C,D, lane 1). The MAbs did not react with PUUV Vranica/Hällnäs N protein used as a negative control (Figure 2C,D, lane 2).

**Figure 3:**
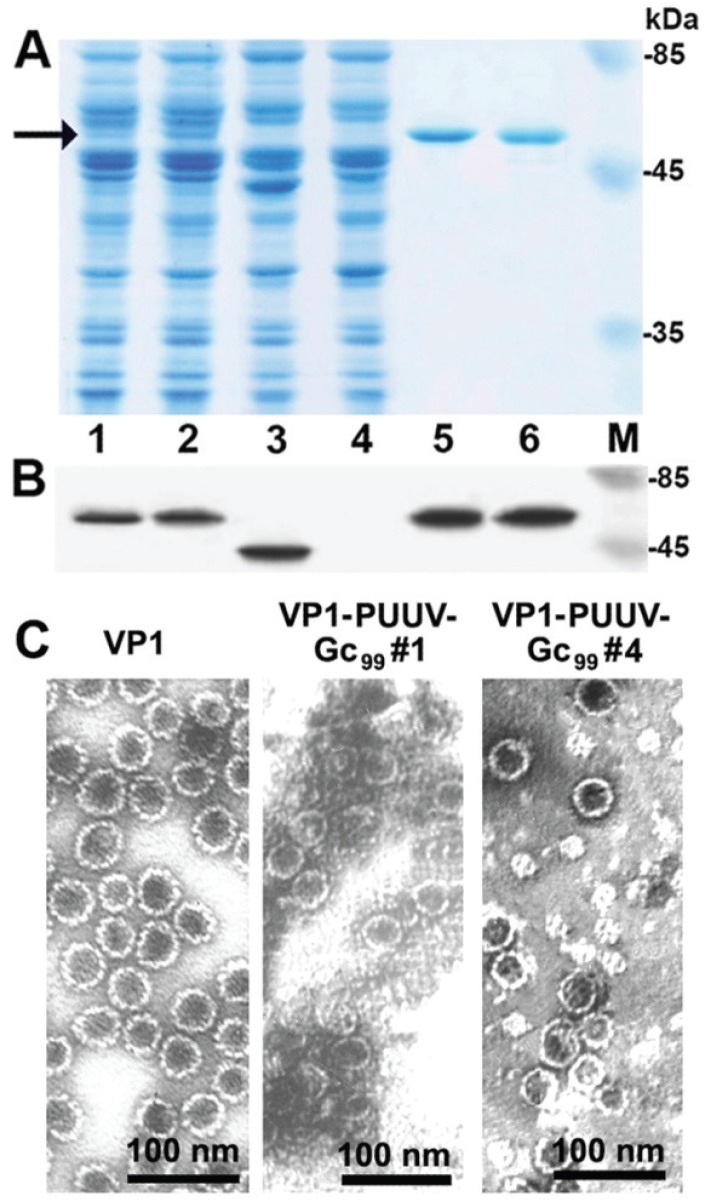
Analysis of VP1-PUUV-Gc_99_ fusion proteins by SDS-PAGE (**A**) and Western blot using VP1-specific MAb 6D11 (**B**). Lane 1, crude lysate of yeast cells transformed with pFX7-VP1/L/Kaz-Gc_99_#1 plasmid; lane 2, crude lysate of yeast cells transformed with pFX7-VP1/L/Kaz-Gc_99_#4 plasmid; lane 3, crude lysate of yeast cells transformed with pFX7-VP1 plasmid; lane 4, negative control sample from crude lysate of *S. cerevisiae* cells transformed with “mock” vector pFX7; lane 5, VP1-PUUV-Gc_99_#1 VLPs after purification in sucrose and CsCl gradients; lane 6, VP1-PUUV-Gc_99_#4 VLPs after purification in sucrose and CsCl gradients; lane M, prestained protein weight marker (UAB “Thermo Fisher Scientific Baltics”); (**C**) Electron microscopy pictures of VP1 and VP1-PUUV-Gc_99_ VLPs stained with 2% aqueous uranyl acetate solution and examined by a JEM-100S electron microscope.

We assumed that the high molecular mass smear observed by Western blot analysis of yeast-expressed Gc protein might be explained by its ubiquitinylation. A Western blot analysis with polyclonal anti-ubiquitin antibody revealed a characteristic pattern of a polyubiquitinylated protein (Figure 2B, lane 1) signifying that indeed yeast-expressed His-tagged PUUV-Gc protein is heavily ubiquitinylated, which is presumably the consequence of an improper folding of this recombinant protein in the yeast cell. Therefore, it is likely that heavily ubiquitinylated recombinant antigen (PUUV-Gc) elicited antibodies against poly-ubiquitin moiety. This may explain the unusual immunostaining pattern with the MAbs #1F5 and #7A12 in Western blot test (Figure 3C,D, lane 1). 

Thus, our attempts to generate Gc-specific MAbs using recombinant full-length Gc protein were unsuccessful.

### 2.2. Expression, Purification and Electron Microscopy Characterization of Chimeric VLPs Harbouring PUUV-Gc Protein Segment

To generate chimeric proteins consisting of hamster polyomavirus (HaPyV) major capsid protein VP1 and PUUV Gc protein segment, yeast *S. cerevisae* were transformed with recombinant plasmids pFX7-VP1/L/Kaz-Gc_99_#1 and pFX7-VP1/L/Kaz-Gc_99_#4. Galactose induction of yeast cells resulted in the production of chimeric proteins VP1-PUUV-Gc_99_#1 and VP1-PUUV-Gc_99_#4 at amounts detectable by SDS-PAGE (Figure 3A, lane 1,2). Both chimeric proteins were found in the soluble fraction of transformed yeast (Figure 3A, lanes 1,2). The identity of chimeric proteins was confirmed by Western blot analysis using VP1-specific MAb #6D11 (Figure 3B, lanes 1,2). 

Recombinant chimeric proteins were purified by sucrose and cesium chloride density gradient centrifugation and detected by SDS-PAGE and Western blot analysis (Figure 3A,B, lanes 3,4). Electron microscopy (EM) analysis of VP1-PUUV-Gc_99_#1 and VP1-PUUV-Gc_99_#4 fusion proteins confirmed the formation of VLPs similar in size and shape to unmodified VP1 VLPs. The diameter of these chimeric VLPs was 45–50 nm, which is typical for polyomavirus capsids (Figure 3C). In addition to normal-sized VLPs, chimeric proteins also formed smaller aggregates that may represent pentamers and their oligomeric variants (Figure 3C).

### 2.3. Antigenic Properties of Chimeric VLPs Harbouring the Gc_99_ Protein Segment

Antigenicity of chimeric VLPs harbouring PUUV-Gc protein segment was investigated by an indirect ELISA using human serum specimens previously found to be positive for PUUV N protein-specific antibodies [63]. The microtiter plates were coated with the VP1-PUUV-Gc_99_ antigens, recombinant yeast-expressed VP1 carrier alone and recombinant full-length PUUV-Gc protein as a positive control. Three out of 9 tested serum specimens positive for PUUV Kazan N protein were reactive with chimeric proteins VP1-PUUV-Gc_99_#1 and VP1-PUUV-Gc_99_#4 (data not shown). The same three serum specimens showed positivity for recombinant full-length PUUV-Gc protein. The reactivity of chimeric VLPs with PUUV-positive human serum specimens confirms surface exposure and accessibility of the inserted Gc sequence. 

### 2.4. Immunogenic Properties of Chimeric VLPs

For immunogenicity testing and generation of Gc-specific MAbs, BALB/c mice were immunized with chimeric proteinsVP1-PUUV-Gc_99_#1 and VP1-PUUV-Gc_99_#4. To achieve a reduced VP1-carrier-specific immune response, subsequent immunizations were performed by alternative use of the two different constructs harbouring the PUUV-Gc protein segment either at position #1 or #4. This approach has been proven successful in our previous study for the generation of the MAbs against a hantavirus N protein segment presented on VLPs [64]. After each immunization, blood specimens of immunized mice were collected and titers of Gc-specific antibodies were monitored by ELISA using full-length PUUV-Gc protein as antigen. Immunization of mice resulted in a strong humoral immune response against the inserted Gc segment: the titers of Gc-specific IgG antibodies in ELISA after the 3rd immunization reached 1–5 × 10^4^ (data not shown). 

### 2.5. Generation of Monoclonal Antibodies against Hantavirus Gc Segment Displayed on VLPs

**Figure 4:**
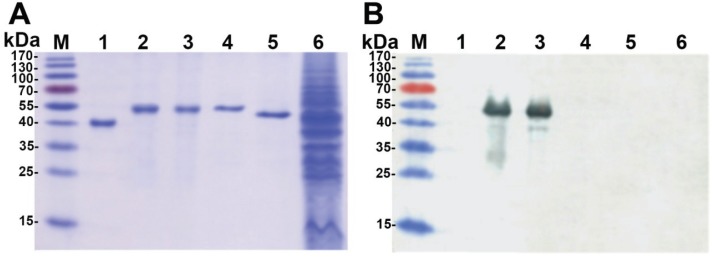
Analysis of the specificity of the MAb #10B8 by Western blot: (**A**) Coomassie blue-stained SDS-PAGE; (**B**) Western blot with MAb #10B8. Lane M, Prestained Protein Molecular Weight Marker (UAB “Thermo Fisher Scientific Baltics”); lane 1, HaPyVP1; lane 2, VP1-PUUV-Gc_99_#1 VLPs; lane 3, VP1-PUUV-Gc_99_#4 VLPs; lane 4, PUUV Kazan N protein; lane 5, Dobrava Slovenia N protein; lane 6, negative control sample from whole crude lysate of *S. cerevisiae* cells transformed with the “mock” vector pFX7.

Spleen cells of a mouse immunized with VLPs harbouring the Gc segment and showing the highest titer of Gc-specific antibodies were used to generate hybridomas. After fusion, all hybrid clones were screened both for reactivity with chimeric VLPs (VP1-PUUV-Gc_99_#1 and VP1-PUUV-Gc_99_#4) and full-length Gc protein. Hybrid clones specific both for chimeric VLPs and PUUV-Gc proteins were subjected to isotyping and recloning. After these procedures, one stable hybridoma clone producing Gc-specific MAb of IgG1 subtype was obtained (clone #10B8). The specificity of #10B8 antibody was proven both by ELISA and Western blot analysis using different yeast-expressed hantavirus antigens. Antibody #10B8 was reactive with recombinant full-length PUUV-Gc protein (data not shown) and chimeric VLPs harbouring PUUV-Gc_99_ segment (Figure 4B lanes 2,3). No reactivity with VP1-carrier, PUUV (strain Kazan) and DOBV (strain Slovenia) N proteins used as negative controls was observed, which confirms the specificity of #10B8 antibody to the Gc sequence (Figure 4B lanes 4,5).

### 2.6. Mapping of the MAb #10B8 Epitope

**Figure 5:**
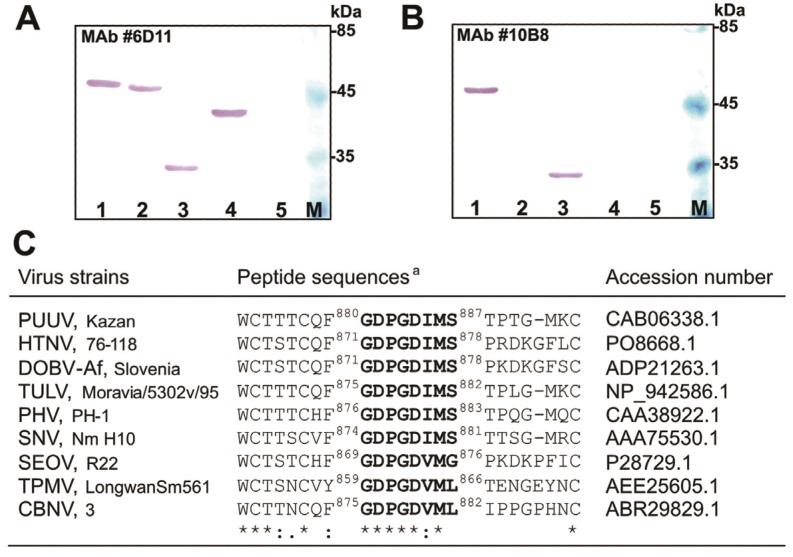
Localization of the epitope recognized by the MAb #10B8. Western blot with the VP1-specific MAb #6D11 (**A**) and Gc-specific MAb #10B8 (**B**). Lane 1, lysate of yeast cells transformed with pFX7-VP1/L/Kaz-Gc_99_#1 plasmid; lane 2, lysate of yeast *S. cerevisiae* cells transformed with pFX7-∆VP1/L/Kaz-Gc_80_#4 plasmid; lane 3, lysate of yeast cells transformed with pFX7-∆VP1/L/Kaz-Gc_17_#1 plasmid; lane 4, lysate of yeast cells transformed with pFX7-VP1 plasmid; lane 5, lysate of yeast cells transformed with “mock” vector pFX7 used as a negative control, lane M, pre-stained protein weight marker (UAB “Thermo Fisher Scientific Baltics”); (**C**) Comparison of the predicted aa sequences for various hantaviruses at the defined binding site of the Gc-specific antibody #10B8. Alignment of the respective sequences of selected Gc proteins was performed using ClustalW program. ^a^ the aa sequences are included with the numbers that represent the aa positions of each peptide.

To identify the epitope recognized by the MAb #10B8, two VP1-derived chimeric proteins harbouring truncated variants of PUUV Kazan Gc protein were constructed (Figure 1). Chimeric proteins were expressed in yeast *S. cerevisiae* and their expression levels were verified by analysing yeast cell lysates by SDS-PAGE and Western blot test with the VP1-specific MAb #6D11 (Figure 5A). Western blot analysis with the #6D11 antibody revealed an efficient expression of both chimeric proteins harbouring truncated Gc protein variants (Figure 5A, lanes 2,3). The Gc-specific MAb #10B8 did not recognize chimeric protein harbouring the 80 aa-long PUUV-Gc segment spanning aa 899–978 of the GPC (construct ΔVP1-PUUV-Gc_80_ #4 in Figure 1; Figure 5B, lane 2). However, it was reactive with the chimeric protein harbouring the 17 aa-long PUUV-Gc segment spanning aa 880–896 of the GPC (construct ΔVP1-PUUV-Gc_17 _#1 in Figure 1; Figure 5B, lane 3). This indicated that the epitope for the MAb #10B8 is located between aa 880 and 896 of the precursor PUUV Gc protein. To identify the epitope more precisely, the reactivity of the MAb #10B8 was analysed by ELISA using a series of synthetic peptides representing the GPC sequences: (#1) GDPGDIMSTPTGMKCPDL (aa 880–897); (#2) GDPGDIMSTPT (aa 880–890) and (#3) GDPGDIMS (aa 880–887). The MAb #10B8 was reactive with all three peptides thus confirming that its epitope is localized in the overlapping 8 aa-long region of hantavirus GPC (aa 880–887). Alignment of Gc protein sequences of different hantaviruses revealed that the sequence spanning aa 880–887 of hantavirus GPC is identical among different hantaviruses, *i.e.*, PUUV, HTNV, DOBV, Tula virus (TULV), Prospect Hill virus (PHV) and Sin Nombre virus (SNV) (Figure 5C).

### 2.7. The Reactivity of the MAb #10B8 with Hantavirus-Infected Cells

**Figure 6:**
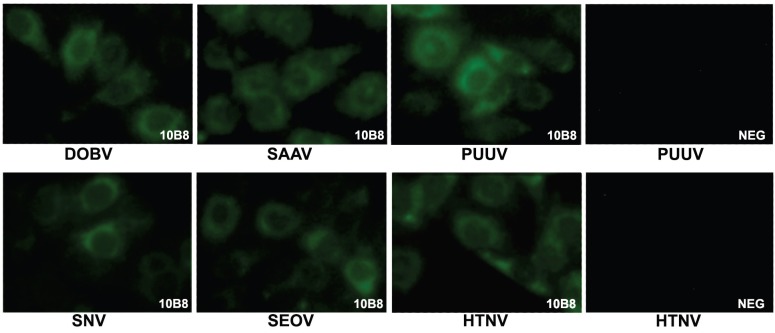
Indirect immunofluorescence staining of PUUV-, Saaremaa virus (SAAV)-, Sin Nombre virus (SNV)-, DOBV-, Seoul virus (SEOV)- and Hantaan virus (HTNV)-infected Vero E6 cells with MAb #10B8. NEG-negative control (PUUV- and HTNV-infected Vero E6 cells incubated with the MAb #2H1 raised against human parainfluenza virus type 3 N protein).

To evaluate the diagnostic potential of the MAb #10B8, its reactivity with hantavirus-infected cells was tested by an immunofluorescence assay (IFA) using commercially available slides. The MAb #10B8 was reactive with PUUV-infected Vero E6 cells and demonstrated a broad cross-reactivity with other hantaviruses, including HTNV, SEOV, DOBV, SNV and Saaremaa virus (SAAV) although there was a slightly different immunoreactivity pattern dependently on hantavirus strain (Figure 6). The strongest immunoreactivity was observed with PUUV whereas the reactivity with HTNV, SEOV, DOBV, SNV and SAAV was slightly lower, which might be explained either by different Gc expression levels in hantavirus-infected cells present in commercial slides, or different accessibility of the MAb epitope in viral Gc proteins (Figure 6). A broad reactivity of the MAb #10B8 with different hantavirus strains is in line with epitope mapping and sequence alignment data that revealed a conserved 8 aa-long sequence (aa 880–887 of PUUV Kazan GPC) as the MAb epitope (Figure 5C).

### 2.8. Neutralization Test with the MAb #10B8

To prove whether the MAb #10B8 has hantavirus-neutralizing activity, it was tested by the chemiluminescence focus reduction neutralization test on PUUV- and DOBV-infected cells. Even at the highest concentrations used, the MAb #10B8 did not cause reduction in the number of foci (data not shown). Therefore, it was concluded that the MAb #10B8 is non-neutralizing.

## 3. Discussion

Antibodies against viral glycoproteins have important diagnostic and therapeutic applications. However, in most cases the MAbs specific to viral glycoproteins are raised against an intact virus or virus-infected cells [41,42,43]. The biosynthesis of viral glycoproteins in heterologous expression systems such as bacteria, yeast, insect or mammalian cells is often problematic due to low expression level, improper folding and limited protein stability [61,62]. In line with previous observations, our attempts to generate MAbs against recombinant full-length PUUV Kazan Gc glycoprotein were unsuccessful. Although we were able to produce full-length PUUV Kazan Gc protein in yeast cells and purify it in amounts sufficient for MAb development, yeast-expressed Gc protein did not induce Gc protein-specific MAbs. Western blot analysis of the recombinant purified PUUV-Gc protein with ubiquitin-specific antibodies revealed a highly ubiquitinylated Gc molecule, which may explain the failure in generation of Gc-specific MAbs. Most probably, the majority of Gc epitopes were hidden by ubiquitin moiety and therefore not accessible for B cells. 

To overcome problems related to low yield and stability of recombinant glycoprotein, we have used an alternative approach for presenting epitopes of hantavirus Gc protein to B cells and inducing an efficient B cell response. We have inserted 99 aa-long segments of PUUV-Gc protein into HaPyV VP1 protein to obtain chimeric VLPs with a surface-exposed Gc_99_ protein sequence. Previous studies have shown that HaPyV VP1-based VLPs tolerate inserts of different size and origin and mediate the induction of high-titered insert-specific antibodies [64,65,66,67,68]. Chimeric VLPs with inserted 120 aa-long segments of hantavirus N protein were successfully used to generate hantavirus-specific MAbs [40,64]. For insertion of a PUUV Kazan Gc protein segment the VP1 positions either #1 (aa 80–89) or #4 (aa 280–289) were selected considering our previous observations that insertion of long protein fragments at positions #1 and #4 less affect VLP formation as compared to potential insert positions #2 (aa 221–224) and #3 (aa 244–246). In addition, insertions into these sites seem to reduce the VP1 immunogenicity [65]. Chimeric proteins harbouring PUUV Gc_99_ segment were efficiently expressed in yeast *S. cerevisiae* and self-assembled to VLPs. The surface exposure and accessibility of the inserted Gc_99_ protein segment was confirmed by the reactivity of native chimeric VLPs with human serum specimens collected from patients seropositive for hantavirus N protein [63]. Chimeric VLPs were reactive with one-third of tested serum specimens, which is in agreement with previous data on the immunodominant role of N protein during hantavirus infection response [18,19,20]. The surface exposure of PUUV Kazan Gc protein sequence inserted into VLPs was also confirmed by the fact that the MAb #10B8 raised against the inserted Gc_99_ segment was reactive with hantavirus-infected cells. Although the selected 99 aa-long segment of PUUV-Gc protein (spanning aa 880–978 of PUUV GPC) contained the epitopes for neutralizing antibodies [20,46] the generated MAb #10B8 did not show any virus-neutralizing activity *in vitro*. This is in line with the epitope mapping of MAb #10B8 at aa 880–887 of PUUV Kazan GPC. This sequence is located outside the previously identified neutralizing epitopes spanning aa 916–924 and aa 954–963 of HTNV GPC [46] as well as aa 918–930 and aa 955–967 of PUUV GPC [20]. On the other hand, although the generated MAb #10B8 is non-neutralizing, it recognizes a highly conserved Gc protein sequence identical among Gc proteins of different hantaviruses. Therefore, the MAb #10B8 is broadly-reactive with different hantavirus strains and represents a promising universal tool for hantavirus detection in infected cells. In a previous report, two neutralizing MAbs that reacted with eight PUUV strains in an immunofluorescence assay have been described. These MAbs did not recognize HTNV, SEOV and PHV, indicating the reactivity with epitopes unique for PUUV. An additional, but non-neutralizing anti-Gc MAb reacted both with PUUV and SEOV. These data indicated the presence of a conserved Gc epitope common for PUUV and SEOV not involved in neutralization [41]. Five human IgG recombinant neutralizing antibodies derived from HTNV-infected patients were able to neutralize HTNV, SEOV and three of them were able to completely neutralize DOBV but not PUUV. Using peptide scans, two partial epitopes of these antibodies, 916-KVMATIDSF-924 and 954-LVTKDIDFD-963, were identified [46].

In conclusion, our study has demonstrated the potential of hamster polyomavirus-derived VP1 protein as a highly efficient carrier for difficult-to-express antigens, such as viral glycoproteins. To our knowledge, this is the first example of a successful expression of chimeric VLPs with inserted large segment of a viral glycoprotein. Chimeric VLPs harbouring surface-exposed glycoprotein segments might be exploited as antigens for detection of glycoprotein-specific antibodies. Moreover, we have demonstrated that chimeric VLPs represent promising immunogens for generation of glycoprotein-specific antibodies.

## 4. Experimental Section

### 4.1. Monoclonal Antibodies and Polyclonal Antisera

The immunodetection was performed using an anti-His MAb (Qiagen, Hilden, Germany), polyclonal rabbit anti-ubiquitin antibody (Abcam, Cambridge, UK), a rabbit antiserum raised against Gc protein segment aa 659–794 of PUUV strain CG18–20 [69] and a control MAb #2H1 directed against parainfluenza type 3 nucleocapsid (N) protein [70]. Human serum specimens (n = 9) were previously collected from patients with suspected hantavirus infection and found to be positive for hantavirus N protein-specific antibodies [63].

### 4.2. Generation of an Expression Plasmid Encoding Full-Length PUUV Gc Glycoprotein

The entire PUUV, strain Kazan, Gc protein-encoding open reading frame (GenBank Acc. No. Z84205.1) was amplified by PCR using pCR-BluntII-TOPO-PUUV-Kaz-Gc plasmid as a template. The PCR amplification was performed according to standard protocols using *Pfu* DNA polymerase (Agilent Technologies, Stratagene, Santa Clara, CA, USA) and using the following primers, containing SpeI restriction sites and six histidine codons (underlined):

5'-TGCACTAGTACAATGCACCATCACCATCACCATGAGACACAGAATTTGAATGCA-3'

5'-GCACTAGTCTAAGGTTTATGCTCTTTTCTG-3'

The obtained PCR amplification product was inserted into the vector pCR-Blunt II-TOPO (Zero Blunt™ TOPO™ PCR Cloning Kit, Life Technologies, Invitrogen, Grand Island, New York, NY, USA). Recombinant plasmids were screened in Top-10 cells (Invitrogen). The hantavirus Gc sequence insert within the pCR-Blunt II-TOPO plasmid was verified by DNA sequencing. The obtained sequence coding for an *N*-terminal hexahistidin (His) tag and the Gc-protein was excised using *Spe* I and subcloned into the *Xba* I site of the yeast expression vector pFX7 [71] under the control of the hybrid GAL10-PYK1 promoter (pFX7-His-PUUV-Kaz-Gc). The pFX7-derived expression plasmid was transformed into the yeast *Saccharomyces cerevisiae* strain 8188 4D (a, *gcn2–284*). As a negative control, the plasmid pFX7 without any insert was used.

### 4.3. Expression and Purification of Recombinant Full-Length Hantavirus Gc and N Proteins

Transformed yeast cells were cultivated and disrupted as previously described [25]. Purification of recombinant PUUV-Gc protein from yeast cell lysate was performed by column affinity chromatography according to manufacturer’s recommendations for denaturing purification of insoluble proteins (The QIAexpressionist, Qiagen, Hilden, Germany). The Gc protein was eluted in buffer E (8 M urea, 0.1 M NaH_2_PO_4_, 0.01 M Tris, pH 4.5). Sodium dodecylsulfate polyacrylamide gel electrophoresis (SDS-PAGE) and Western blot analysis were accomplished to prove the identity and purity of the eluted recombinant Gc protein.

N proteins of PUUV, strains Kazan and Sotkamo, of DOBV, genotype Dobrava strain Slovenia, and Kurkino strain SK/Aa, and of HTNV, strain Fojnica, were purified as previously described [25].

### 4.4. Generation of Expression Plasmids Encoding a VP1 Gc Fusion Protein

A DNA fragment encoding a segment of PUUV, strain Kazan Gc protein spanning aa residues 880–978 was amplified by PCR using pCR-Blunt II-TOPO-PUUV-Kaz-Gc as template and using the following primers, containing *Bam*H I restriction sites (underlined):

Kaz-Gc-BD 5'-GCGGATCCTGGAGATATAATGAGCACAC-3'

Kaz-Gc-RB 5'-GCGGATCTGTTTCACTTAAATCTTG-3'

The PCR product was inserted into the *Bgl* II cloning site within the gene encoding hamster polyomavirus (HaPyV) major capsid protein VP1 either at position #1 (codons 80–89) or position #4 (codons 280–289) [68]. This cloning strategy resulted in plasmids pFX7-VP1/L/Kaz-Gc_99_#1 and pFX7-VP1/L/Kaz-Gc_99_#4 where the PUUV-Kazan-Gc_99_ protein-encoding sequence is inserted either at position #1 or #4, and flanked by GSSG-encoding linkers (L) [67]; see Figure 1. Plasmids pFX7-∆VP1/L/Kaz-Gc_17_#1 and pFX7-∆VP1/L/Kaz-Gc_80_#4 used for MAb #10B8 eptope mapping were created after removing part VP1 and PUUV-Gc_99_-encoding sequence by cleavage with restriction enzymes *Smi* I and *Acc* 65I, blunting and religation of plasmid pFX7-VP1/L/Kaz-Gc_99_#1 and by cleavage with *Smi* I and *Bsp* 119, blunting and religation of plasmid pFX7-VP1/L/Kaz-Gc_99_#4, respectively (Figure 1).

### 4.5. Expression and Characterization of Chimeric VP1 Protein Harbouring PUUV Gc Protein Insert

The expression of the VP1 and VP1-PUUV-Gc fusion proteins in yeast *S. cerevisiae* strain AH22–214 (a, *leu2, his4*) and their purification was performed as described previously for VP1 and other chimeric proteins [68,72]. The purified VP1-PUUV-Gc proteins were stored at −20 °C in 50% glycerol and samples of purified recombinant VP1-PUUV-Gc_99_ proteins were placed on 200-mesh carbon coated Cu grids, negatively stained with 2% aqueous uranyl acetate solution and examined by JEM-100S electron microscope (JEOL, Tokyo, Japan). For comparison, authentic HaPyV-VP1-derived VLPs were examined. 

### 4.6. SDS-PAGE and Western Blot Analysis

Purified proteins (100 ng/lane) or crude cell lysates (1 μg of total protein per lane) were fractionated by SDS-PAGE in 10% mini-gels. Proteins were detected in the SDS PAGE by Coomassie blue staining (Sigma-Aldrich Co, St. Louis, MO, USA). For Western blot, separated proteins were transferred to polyvinyldifluoride (PVDF) membranes (Roth, Karlsruhe, Germany) under semidry conditions. Membranes were blocked by incubation in 5% dry milk/phosphate-buffered saline (PBS) for 1 h at RT, washed in PBS containing 0.1% Tween 20 (PBS-T) and incubated for 1 h at RT with the respective primary antibodies. His-tagged Gc protein was detected using anti-tetra-His MAb (Qiagen, Hilden, Germany) and rabbit anti-PUUV Gc protein antibodies. VP1 and chimeric VP1-derived proteins were detected using the MAb #6D11 raised against HaPyV VP1 [64]. The potential ubiquitinylation of the recombinant Gc protein was analysed using anti-ubiquitin rabbit polyconal antibody (Abcam, Cambridge, UK). Prior to incubation with the anti-ubiquitin antibody, the membrane was autoclaved in deionized water for 20 min, as previously recommended [73]. For testing MAb specificity, the membranes were incubated with undiluted hybridoma supernatant. After incubation with primary antibodies and washing in PBS-T, the membranes were incubated for 1 h at RT with the respective secondary antibodies labeled with horse-radish peroxidase (HRP): either anti-mouse IgG (Bio-Rad, Hercules, CA, USA 1:5,000) or anti-rabbit IgG (Bio-Rad, Hercules, CA, USA). The blots were visualized using 3',5,5'-tetramethylbenzidine (TMB) blotting *ready-to-use* substrate (Sigma-Aldrich Co., St. Louis, MO, USA).

### 4.7. Immunization of Mice and Generation of Monoclonal Antibodies

All procedures involving laboratory mice were performed at the breeding colony of the Center for Innovative Medicine (Vilnius, Lithuania) under controlled laboratory conditions in strict accordance with Lithuanian and European legislation. 

To generate MAbs against the recombinant PUUV-Gc protein, three BALB/c mice were injected subcutaneously 3 times with 50 µg of recombinant PUUV-Gc protein at 21-day intervals. To generate the MAbs against the PUUV-Gc segment inserted into VLPs, the 1st and the 3rd immunizations were done with VP1-PUUV-Gc_99_#1 protein, the 2nd immunization was done with VP1-PUUV-Gc_99_#4 protein. For a primary immunization, the antigen was emulsified in Complete Freund’s adjuvant (Sigma-Aldrich Co.), the subsequent immunizations were performed with antigens dissolved in PBS without adjuvant. After the 3rd immunization, serum specimens were collected to determine the titers of specific antibodies. Finally, the mouse with the highest anti-Gc titer was boosted with the same dose of VP1-PUUV-Gc_99_#4 protein in PBS and sacrificed for hybridoma development. 

Hybridomas were generated essentially as described previously [74]. Four days after the booster immunization, spleen cells of the immunized mouse were fused with mouse myeloma Sp2/0 cells using PEG 1500 as a fusion agent (PEG/DMSO solution, HybriMax, Sigma-Aldrich Co.). Hybrid cells were selected in growth medium supplemented with hypoxanthine, aminopterin and thymidine (50× HAT media supplement, Sigma-Aldrich Co.). Viable clones were screened by an indirect ELISA using 96-well microtiter plates coated with the respective antigens, either full length PUUV-Gc protein or VP1-PUUV-Gc_99_#1 or VP1-PUUV-Gc_99_#4 protein. Positive clones were stabilized by limiting dilution cloning on a macrophage feeder layer. Hybridoma cells were maintained in complete Dulbecco’s modified Eagle’s medium (DMEM) containing 15% fetal calf serum (FCS, Biochrom, Cambridge, UK) and antibiotics. Heavy chain types of MAbs were determined by ELISA using Monoclonal Antibody Isotyping Kit (ISO-2, Sigma-Aldrich Co.). 

### 4.8. Indirect ELISA

The specificity of mouse antisera, MAbs and human sera was tested by an indirect ELISA. Microtiter plates (Nunc MaxiSorp, Roskilde, Denmark) were coated with the respective antigens by adding 100 μL of the antigen solution (2 μg/mL) in coating buffer (50 mM sodium carbonate, pH 9.5) and incubation overnight at 4 °C. The plates were blocked for 30 min at RT with 1% bovine serum albumin (BSA) in PBS and then incubated with either serially diluted serum sample or undiluted hybridoma supernatant for 1 h at RT. After washing, the plates were incubated with HRP-labeled anti-mouse IgG (Bio-Rad, 1:5,000 in PBS-T) or anti-human IgG (Bio-Rad) for 1 h at RT. The immune reaction was detected with TMB *ready-to-use* substrate (Sigma-Aldrich Co.). The optical density (OD) was measured at 450 nm in a microtiter plate reader (Tecan, Groedig, Austria). 

Epitope mapping was performed by an indirect ELISA using biotinylated synthetic peptides (Genscript, Piscataway Township, New Jersey, USA) harbouring PUUV Kazan GPC sequences (underlined) aa 880–897 (#1), aa 880–890 (#2) or aa 880–887 (#3): (#1) biotin-SGSGGDPGDIMSTPTGMKCPDL-OH; (#2) biotin-SGSGGDPGDIMSTPT-OH; (#3) biotin-SGSGGDPGDIMS-OH. Peptide harbouring human hepatitis B virus surface protein sequence (underlined) aa 116–127 (biotin-SGSGSTGPCRTCTTPA-OH) was used as a negative control. To test the reactivity of MAb with biotinylated synthetic peptides, the microtiter plates (Nunc MaxiSorp, Nalge Nunc Intl., Penfield, NY, USA) were coated with 100 μL of avidin (Amresco, Solon, OH, USA) at 5 μg/mL in deionized water and air-dried overnight at 37 °C. After washing, 100 μL of biotinylated peptides (10 μg/mL) were added to the avidin-coated wells and incubated for 1 h at 37 °C. The plates were then incubated with 100 μL of undiluted hybridoma supernatant and developed as above.

### 4.9. Indirect Immunofluorescence Assay

Indirect immunofluorescence assay (IFA) was performed using commercial IFA slides with PUUV (strain Sotkamo)-, DOBV (strain Slovenia)-, Saaremaa virus-, SEOV-, HTNV- and SNV-infected Vero E6 cells (EuroImmun, Lübeck, Germany) as recommended by the manufacturer. Briefly, cell culture supernatant of hybridoma #10B8 was added to the slides in a dilution of 1:50 in PBS or undiluted. The negative control MAb #2H1directed against parainfluenza type 3 N protein [70] was used at the same dilution. Detection was done by incubation with a fluorescein isothiocyanate (FITC)-conjugated goat anti-mouse immunoglobulin (H+L) IgG (DakoCytomation, Glostrup, Denmark) diluted 1:20 in PBS. Cells were counter-stained with Evans Blue and IFA slides were analyzed using a Olympus IX-70 fluorescence microscope (Olympus, Tokyo, Japan) with 40× objective at an excitation of 495 nm and Image-pro Plus Version 7.0 [75]. 

### 4.10. Hantavirus Neutralization Test

Hantavirus-neutralizing activity of the MAb #10B8 was investigated using the chemiluminescence focus reduction neutralization test (c-FRNT) essentially as described previously [76]. Hybridoma #10B8 supernatant was first diluted serially starting at 1:4, mixed with an equal volume containing 30–80 focus forming units (FFU) of the virus (PUUV, strain Sotkamo, or DOBV, genotype Kurkino, strain SK/Aa) and incubated for 1 h at 37 °C prior inoculating the cells. After 6–10 days of incubation, PUUV- and DOBV-N specific polyclonal rabbit antisera, followed by peroxidase-labeled goat anti-rabbit IgG and chemiluminescence substrate Super Signal West Dura (Pierce, Rockford, IL, USA) were used to detect the viral antigen in infected cells. At least 80% reduction in the number of foci was considered as the criterion for virus neutralization. 

## 5. Conclusions

Hamster polyomavirus VP1 protein-derived chimeric VLPs harboring a 99 aa-long segment of PUUV Gc glycoprotein were efficiently produced in yeast and used to generate a Gc-specific MAb that reacts with hantavirus-infected cells. The generated MAb #10B8 recognizes *N*-terminally located Gc epitope (aa 880–887 of GPC) conserved among different hantavirus strains. The current study demonstrates the potential of hamster polyomavirus-derived VP1 protein as a carrier for segments of difficult-to-express antigens, such as viral glycoproteins. The generated broadly-reactive MAb #10B8 might be useful for various diagnostic applications.
